# Development and Validation of Predictive Model—HASBLAD Score—For Major Adverse Cardiovascular Events During Perioperative Period of Non-cardiac Surgery: A Single Center Experience in China

**DOI:** 10.3389/fcvm.2022.774191

**Published:** 2022-05-09

**Authors:** Menglin Zhao, Zhi Shang, Jiageng Cai, Cencen Wu, Yuan Xu, Lin Zeng, Hong Cai, Mao Xu, Yuanyuan Fan, Yanguang Li, Wei Gao, Weixian Xu, Lingyun Zu

**Affiliations:** Department of Cardiology, Institute of Vascular Medicine, Peking University Third Hospital, Beijing, China

**Keywords:** non-cardiac surgery, major adverse cardiovascular events, prediction model, Takotsubo cardiomyopathy, nomogram, risk score

## Abstract

**Background:**

Major adverse cardiovascular events (MACEs) represent a significant reason of morbidity and mortality in non-cardiac surgery during perioperative period. The prevention of perioperative MACEs has always been one of the hotspots in the research field. Current existing models have not been validated in Chinese population, and have become increasingly unable to adapt to current clinical needs.

**Objectives:**

To establish and validate several simple bedside tools for predicting MACEs during perioperative period of non-cardiac surgery in Chinese hospitalized patients.

**Design:**

We used a nested case-control study to establish our prediction models. A nomogram along with a risk score were developed using logistic regression analysis. An internal cohort was used to evaluate the performance of discrimination and calibration of these predictive models including the revised cardiac risk index (RCRI) score recommended by current guidelines.

**Setting:**

Peking University Third Hospital between January 2010 and December 2020.

**Patients:**

Two hundred and fifty three patients with MACEs and 1,012 patients without were included in the training set from January 2010 to December 2019 while 38,897 patients were included in the validation set from January 2020 and December 2020, of whom 112 patients had MACEs.

**Main Outcome Measures:**

The MACEs included the composite outcomes of cardiac death, non-fatal myocardial infarction, non-fatal congestive cardiac failure or hemodynamically significant ventricular arrhythmia, and Takotsubo cardiomyopathy.

**Results:**

Seven predictors, including **H**emoglobin, C**A**RDIAC diseases, Aspartate aminotransferase (A**S**T), high **B**lood pressure, **L**eukocyte count, general **A**nesthesia, and **D**iabetes mellitus (HASBLAD), were selected in the final model. The nomogram and HASBLAD score all achieved satisfactory prediction performance in the training set (*C* statistic, 0.781 vs. 0.768) and the validation set (*C* statistic, 0.865 vs. 0.843). Good calibration was observed for the probability of MACEs in the training set and the validation set. The two predictive models both had excellent discrimination that performed better than RCRI in the validation set (*C* statistic, 0.660, *P* < *0.05* vs. nomogram and HASBLAD score).

**Conclusion:**

The nomogram and HASBLAD score could be useful bedside tools for predicting perioperative MACEs of non-cardiac surgery in Chinese hospitalized patients.

## Introduction

With the continuous improvement of human life expectancy, increasing number of elderly patients underwent surgery (1). A considerable proportion of these patients present with cardiovascular risk factors or suffer from cardiovascular comorbidities, leading to an increased risk of perioperative cardiovascular complications. Major adverse cardiovascular events (MACEs) represented a significant source of perioperative morbidity and mortality (2). Up to 42% of perioperative deaths were due to cardiac complications, such as myocardial infarction (MI), cardiac arrest, and congestive heart failure (HF) (3), and the incidence of these complications was likely to rise in the aging population (4). The prevention of perioperative cardiac complications has been one of the hotspots in the research field, which requires comprehensive preoperative risk assessment and postoperative monitoring of patients at risk.

Current guidelines highly recommended the use of predictive models to assess the risk of perioperative MACEs (1, 5–7), including the revised cardiac risk index (RCRI) (8), and the American College of Surgeons National Surgical Quality Improvement Program (NSQIP) risk model (9). RCRI was the most widely validated and used model. However, as an index that has existed for over two decades, it has limitations to meet the clinical requirement, including the underestimation of the cardiac risk (10), and inadequate representation of high-risk subgroups (8). With respect to the NSQIP model, although the internal validation showed excellent predictive ability, with a *C*-statistic of 0.895 for predicting cardiovascular complications, none of the NSQIP-derived calculators have been robustly externally validated. Moreover, it was too complicated to use at the bedside (11).

In China, its huge and aging population, expanding economy, and lifestyle changes have created a tremendous demand for perioperative healthcare services. Nearly 20 million elderly patients-−25% of all patients—need surgery in China each year (12).

However, there was no predictive model based on Chinese data, and the predictive models recommended by the guidelines have not been validated in the Chinese population.

Therefore, we sought to establish and validate an effective risk predictive model based on the Chinese population, hoping to provide convenient bedside tools for predicting perioperative MACEs with satisfactory performance in non-cardiac surgeries.

## Materials and Methods

### Study Design

This is a single-center, observational study conducted in accordance with the Transparent Reporting of a Multivariable Prediction Model for Individual Prognosis or Diagnosis statement (13). The selection of the training set was based on a nested case-control study, while the selection of the validation set was based on a cohort study.

### Study Population and Data Collection

**Figure 1:**
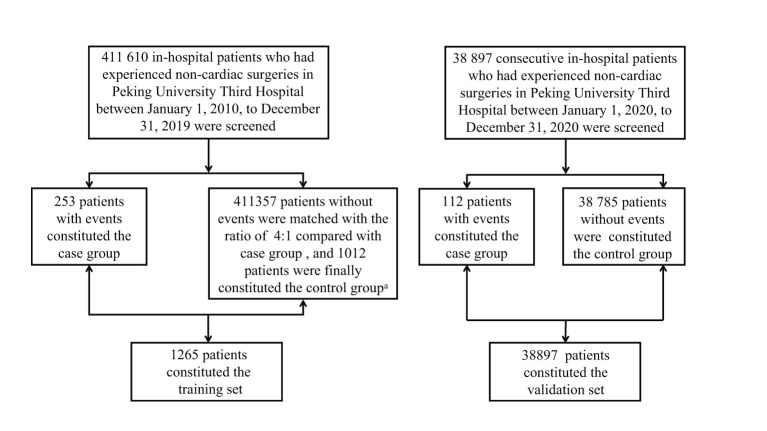
Patients screening process of training set and validation set. a: The patients were matched according to the type of operation, operative time and age with the ratio of 4:1 between non-events group and events group. The matched patients belonged to one same operation type which was demonstrated in Supplementary Table 1 in Supplemental Appendix, while they were randomly selected in patients who had operation one month before or after the patients in events group.

We conducted a retrospective review of all patients who underwent in-hospital non-cardiac surgeries with age over 18 years by a medical record system in the Peking University Third Hospital between January 2010 and December 2019. We selected surgical types proposed in the 2014 ESC guidelines (Supplementary Table 1). Non-cardiac surgeries contained the operations conducted on organs except for the heart and its appendages (the details were showed in Supplementary Table 1). The operation risk level of surgical operation ranged from low to high. Then we matched the patients with the same age who underwent the same type of surgery at the same time (1 month before or after the operation of patients with MACEs) who had no MACEs, as the control group, according to the ratio of 1:4 (Figure 1). With respect to the validation set, we prospectively selected patients who underwent non-cardiac surgery with age over 18 years from January 2020 to December 2020. Since all of patients who suffered perioperative MACEs would need to apply for multidisciplinary consultation including cardiology, the information of application records, diagnosis and treatment experience could be recorded. Through the multidisciplinary consultation system, we could monitor the situation for perioperative complications of patients timely and collect the exact number of patients broken out the cardiovascular events without obvious omissions. At the same time, the number of surgeries for the whole year of 2020 was extracted from the medical record system.

The International Classification of Diseases, Tenth Revision (ICD-10) has been used to extract the target population. Subsequently, trained clinical reviewers collected clinical data from the medical chart, operative archives, anesthesia records, and progress notes with the surgical attending. Then, we collected demographic data, co-morbidities, laboratory results, inspection status, and procedure-related data, including the type of surgery, procedure risk, and length of stay for each patient. Cardiac diseases included acute and chronic HF, chronic coronary disease, acute coronary syndrome, severe aortic stenosis, severe aortic regurgitation, severe mitral valve stenosis, severe mitral valve regurgitation, atrial fibrillation, ventricular tachyarrhythmias, and patients with pacemaker/implantable cardioverter defibrillator. Venous blood samples were drawn in the fasting condition to test leukocyte, hemoglobin (HGB), platelet (PLT), alanine aminotransferase (ALT), aspartate aminotransferase (AST), serum creatinine, potassium, and fibrinogen.

This study was conducted in accordance with the Declaration of Helsinki and with approval from the ethics committee of Peking University Third Hospital (No. M2018258).

### Study Outcomes

The MACEs included the composite outcomes of cardiac death, non-fatal MI, non-fatal congestive cardiac failure, hemodynamically significant ventricular arrhythmia, and Takotsubo cardiomyopathy of patients who underwent non-cardiac surgeries during hospitalization. Cardiac death was defined as sudden death or death secondary to MI, arrhythmia, or HF. MI was defined according to the Fourth universal definition of ESC guideline (14). Hemodynamically significant ventricular arrhythmia included ventricular tachycardia, ventricular flutter, and fibrillation that could affect hemodynamics apparently. Takotsubo cardiomyopathy was defined based on diagnosis criteria of Mayo Clinic (15).

### Statistical Analysis

Statistical analysis was performed using R 4.0.3. We first classified the patients with MACEs in the training set according to the operation types (a total of 30) proposed in the 2014 ESC guidelines (Supplementary Tables 1, 2). Then, among the patients without MACEs in the training set, the process of matching was conducted using propensity score matching (PSM). One patient with MACE matched 4 patients without MACEs using operation type (showed in Supplementary Table 1), age, and operation time (particular year of operation). Continuous variables conformed to the normal distribution were expressed as the mean ± standard deviation, and t-test was used for comparison between two groups. While medians (Interquartile range) and Wilcoxon tests were used for continuous variables not conformed to normal distribution. Categorical variables were presented as percentages with Chi-squared test or Fisher's exact test for comparison between two groups. We used univariable logistics regression to select variables firstly entering to the regression model with P-value < 0.1. Then multivariable logistic regression analysis was conducted by a backward stepwise method to choose variables ultimately be included in the model. The variables included in the model were used to construct the nomogram using the package “rms.” To construct a risk score, restricted cubic spline (RCS) was used to help finding the best cut-off values of continuous variables. The multivariable logistic regression model was performed with all variables which were categorical. We used odds ratio (OR) as the weight to assign a value to each variable. The area under the curve (AUC) for the receiver operating characteristic (ROC) curve was used to validate the discrimination efficiency of the model and a fifth quantile calibration curve was used to assess the model's calibration (16). The Delong test was used to evaluate whether the difference between the two ROC curves was statistically significant. The local institutional review board approved this analysis.

## Results

### Study Population and Baseline Characteristics

After the screening period, there were 1,265 patients (MACEs group: 253; non-MACEs group: 1,012) in the training set and 38,897 patients (MACEs group: 112; non-MACEs: 38,785) in the validation set. The specific screening process was shown in Figure 1.

In the training set, patients with the age older than 70 years accounted for 51.5% (129 patients). There was no significant difference in age (median age: 71 vs. 70.5 years, P = 0.497) and sex (males, 43.9 vs. 47.5%, P = 0.331) between the two groups. The prevalence of cardiac diseases (35.2 vs. 12.1%, P < 0.001), hypertension (HT) (55.3 vs. 37%, P < 0.001), diabetes mellitus (DM) (29.2 vs. 11.4%, P < 0.001), chronic kidney disease (CKD) (5.5 vs. 1.9%, P = 0.002), and general anesthesia (72.3 vs. 64.9%, P = 0.031) was significantly higher in patients with MACEs. There were less endoscopic surgeries in MACEs group (18.2 vs. 31.7%, P < 0.001). Leukocyte, ALT, AST, and fibrinogen levels were significantly higher in MACEs group, whereas HGB and PLT levels were significantly lower. Baseline characteristics of the training set were presented in Table 1.

**Table 1:** Baseline characteristics of the training set.

**Variables**	**MACEs** **(*n* = 253)**	**Non-MACEs** **(*n* = 1012)**	***P*-value**
Age (years)	71 (58, 78)	70.5 (58, 78)	0.497
≤70	124 (49%)	506 (50%)	
>70	129 (51%)	506 (50%)	
Sex (males)	111 (43.9%)	481 (47.5%)	0.331
Endoscopic operation	46 (18.2%)	321 (31.7%)	<0.001
General anesthesia	183 (72.3%)	657 (64.9%)	0.031
Length of stay (days)	14 (9, 22)	9 (5,15)	<0.001
Cardiac disease	89 (35.2%)	122 (12.1%)	<0.001
HT	140 (55.3%)	374 (37%)	<0.001
DM	74 (29.2%)	115 (11.4%)	<0.001
CKD	14 (5.5%)	19 (1.9%)	0.002
Leukocyte (×109 l^−^1)	7.8 (6.23, 10.79)	6.48 (5.35, 8.1)	<0.001
HGB (g l^−^1)	120 (102, 136)	131 (118, 143)	<0.001
PLT (×109 l^−^1)	191 (150, 245)	206 (172.75, 255)	0.001
ALT (U l^−^1)	24 (15, 39)	16 (12, 24)	<0.001
AST (U l^−^1)	24 (17, 40)	21 (17, 25)	<0.001
Scr (mmol l^−^1)	81 (66, 108)	74 (63, 88)	<0.001
Potassium (mmol l^−^1)	4.08 (3.8, 4.42)	4.05 (3.81, 4.3)	0.258
Fibrinogen (g l^−^1)	3.43 (2.79, 4)	3.14 (2.69, 3.71)	0.001

*HT, hypertension; DM, diabetes mellitus; CKD, chronic kidney disease; HGB, hemoglobin; PLT, platelet; ALT, alanine aminotransferase; AST, aspartate aminotransferase; Scr, serum creatinine*.

### Major Adverse Cardiovascular Events

Two hundred and fifty three patients in the training set suffered MACEs during the perioperative period. The composition of cardiovascular outcomes was as follows: cardiac death (81; 31.7%), nonfatal HF (78; 31%), nonfatal MI (71; 28.2%), hemodynamically arrhythmia (14; 5.6%), and Takotsubo cardiomyopathy (9; 3.5%). There were 112 patients suffered MACEs in the validation set, with the proportion of MACEs as follows: cardiac death (10; 8.9%), HF (69; 61.6%), nonfatal MI (21; 18.8%), hemodynamically arrhythmia (3; 2.7%), and Takotsubo cardiomyopathy (9; 8.0%). Details were illustrated in Table 2.

**Table 2:** The proportion of MACEs in both training set and validation set.

**MACEs**	**Training set** **(n = 253)**	**Validation set** **(n = 112)**
Cardiac death	81 (31.7%)	10 (8.9%)
HF	78 (31.0%)	69 (61.6%)
Non-fatal MI	71 (28.2%)	21 (18.8%)
Hemodynamically arrhythmia	14 (5.6%)	3 (2.7%)
Stress cardiomyopathy	9 (3.5%)	9 (8.0%)

*MACEs, major adverse cardiovascular events; HF, heart failure; MI, myocardial infarction*.

### Development of the Prediction Model of Nomogram

**Figure 2:**
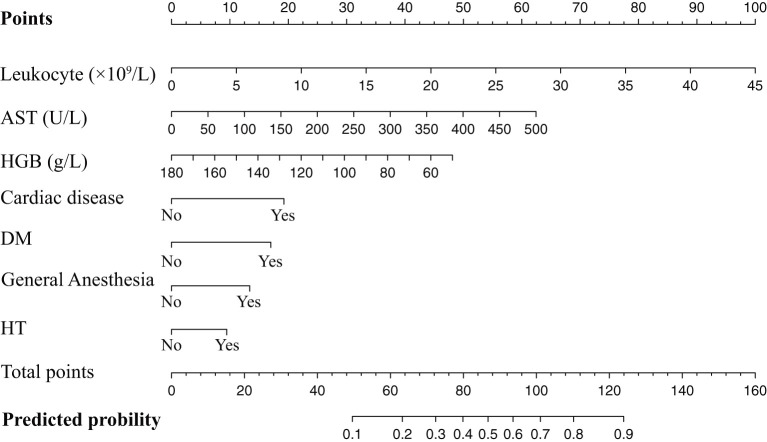
Nomogram of the training set used to predict the MACEs risk. AST, aspartate aminotransferase; HGB, hemoglobin; DM, diabetes mellitus; HT, hypertension.

The results of the univariate logistic regression analysis were shown in Table 3. Cardiac disease, HT, DM, CKD, endoscopic operation, length of hospital stay, general anesthesia, leukocyte, HGB, PLT, ALT, AST, and fibrinogen were significantly associated with perioperative MACEs with the preset standard of P < 0.1, which would be included in multivariate analysis preliminarily. After considering the clinical practice and results of logistic regression, we finally selected seven factors for Model 1, including cardiac diseases, HT, DM, general anesthesia, leukocyte, HGB, and AST. We also illustrated the Model 1 in the form of a nomogram for more convenient use in clinical work. In the nomogram, the score assigned to each variable was proportional to its risk contribution to MACEs. The total score on the risk axis represented the probability of MACEs risk. The higher the score, the higher the risk of developing MACEs in the perioperative period. Details were showed in Table 3 and Figure 2.

**Table 3:** Results of univariate logistic regression analysis.

**Variables**	**OR**	**95% CI**	**P-value**
Age	1.002	0.994-1.011	0.566
Sex (vs. female)	1.159	0.878-1.529	0.297
Endoscopic operation	0.478	0.339-0.676	<0.001
General anesthesia	1.413	1.042-1.915	0.026
Length of stay	1.048	1.035-1.061	<0.001
Cardiac disease	3.959	2.875-5.452	<0.001
HT	2.113	1.599-2.793	<0.001
DM	3.225	2.311-4.500	<0.001
CKD	3.061	1.513-6.194	0.002
Leukocyte	1.163	1.119-1.210	<0.001
HGB	0.976	0.969-0.982	<0.001
PLT	0.998	0.996-1.000	0.081
ALT	1.01	1.005-1.014	<0.001
AST	1.01	1.006-1.015	<0.001
Scr	1.001	1.000-1.002	0.105
Potassium	1.2	0.890-1.619	0.231
Fibrinogen	1.231	1.084-1.398	0.001

*OR, odds ratio; CI, confidence interval; HT, hypertension; DM, diabetes mellitus; CKD, chronic kidney disease; HGB, hemoglobin; PLT, platelet; ALT, alanine aminotransferase; AST, aspartate aminotransferase; Scr, serum creatinine*.

### Converting Continuous Variables to Categorical Ones

**Figure 3:**
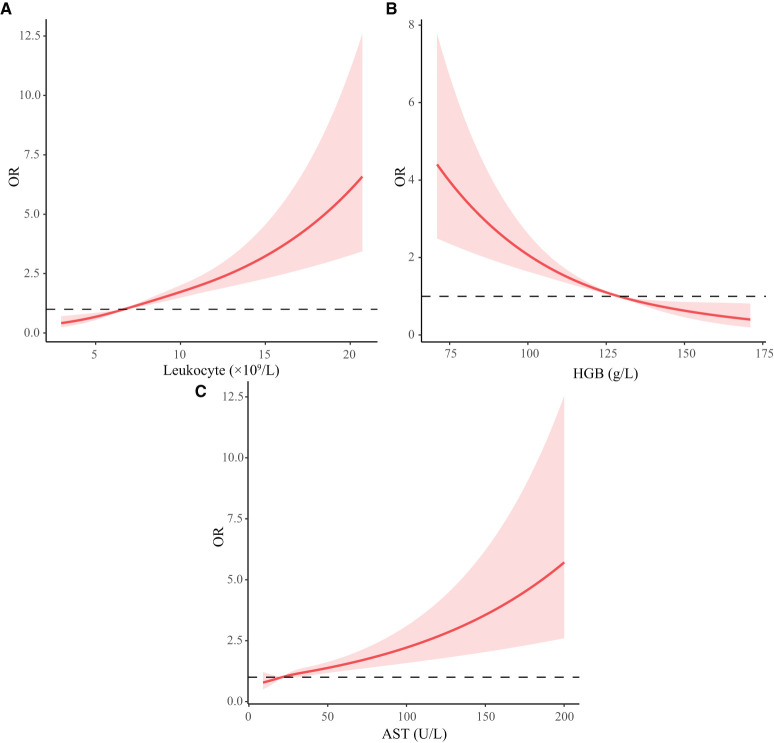
Restricted cubic spline curve of continuous variables. HGB, hemoglobin; AST, aspartate aminotransferase; ALT, alanine aminotransferase. **(A-C)** Represents the changing trend of risk of MACEs with leukocyte, HGB, and AST.

We used RCS curves to evaluate the relationship between continuous variables, including leukocyte, HGB and AST, and MACEs. Leukocyte and AST were positively correlated with MACEs, while HGB was negatively correlated with MACEs. Combined with clinical experience and the results of statistical analysis, we take 10 × 10^9^ /L, 110 g/L, and 40 U/L as cut-off points of leukocyte, HGB, and AST respectively, converting them to categorical variables. Details were illustrated in Table 4 and Figure 3.

**Table 4:** Results of multivariate logistic regression analysis in the training set.

**Variables**	**Model 1 (nomogram)**		**Variables**	**Model 2**	
	**β**	**OR (95% CI)**		**β**	**OR (95% CI)**
DM			DM		
No		Reference	No		Reference
Yes	1.005	2.732 (1.855-4.025)	Yes	1.025	2.788 (1.889-4.115)
Cardiac disease			Cardiac disease		
No		Reference	No		Reference
Yes	1.138	3.119 (2.171-4.482)	Yes	1.143	3.135 (2.179-4.511)
HT			HT		
No		Reference	No		Reference
Yes	0.559	1.749 (1.260-2.426)	Yes	0.564	1.757(1.265-2.441)
Leukocyte	0.131	1.140 (1.094-1.189)	Leukocyte		
			≤10 × 10^9^ l^−1^		Reference
			>10 × 10^9^ l^−1^	0.620	1.858 (1.281-2.696)
HGB	−0.022	0.978 (0.971-0.986)	HGB		
			≥110 g l^−1^		Reference
			<110 g l^−1^	0.961	2.615 (1.828-3.739)
AST	0.007	1.007 (1.003-1.011)	AST		
			≤40 U l^−1^		Reference
			>40 U l^−1^	1.677	5.352 (3.424-8.365)
General anesthesia			General anesthesia		
No		Reference	No		Reference
Yes	0.792	1.207 (1.538-3.168)	Yes	0.696	2.006 (1.398-2.879)

*OR, odds ratio; CI, confidence interval; DM, diabetes mellitus; HT, hypertension; HGB, hemoglobin; AST, aspartate aminotransferase*.

### Construction of Risk Score

We then built Model 2 with the same seven risk factors in the training set, after converting leukocyte, HGB, and AST to categorical variables. Using OR of model 2 for the weights of assigning scores, we defined OR ≥ 2 as an independent high-risk factor and assigned 2 points, including cardiac diseases, general anesthesia, DM, AST (> 40 U/L), and HGB (<110 g/L). For risk factors with OR <2, 1 point shall be assigned including leukocyte (>10 × 10^9^/L) and HT, constructing the HASBLAD score. If there were no above risk factors, 0 points will be assigned. The complete scoring table was shown in Table 5.

**Table 5:** Score assignment of model variables.

**Variables**	**OR**	**Points assigned**
HGB (<110 vs. ≥110 g l^−1^)	2.615	2
CARDIAC disease (yes vs. no)	3.135	2
AST (>40 vs. ≤40 U l^−1^)	5.352	2
Blood pressure (HT, yes vs. no)	1.757	1
Leukocyte (>10 vs. ≤10 × 10^9^ l^−1^)	1.858	1
General anesthesia (yes vs. no)	2.006	2
DM (yes vs. no)	2.788	2

*DM, diabetes mellitus; HT, hypertension; AST, aspartate aminotransferase; HGB, hemoglobin*.

### Evaluation of Model Discrimination and Calibration in the Training Set

**Figure 4:**
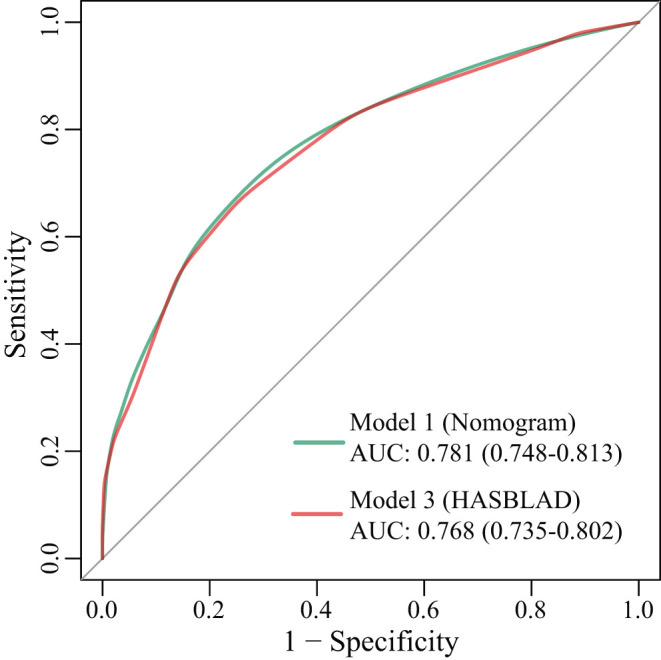
ROC curves for Model 1, Model 3 in the training set. AUC, area under the curve; ROC, receiver operating characteristic.

**Figure 5:**
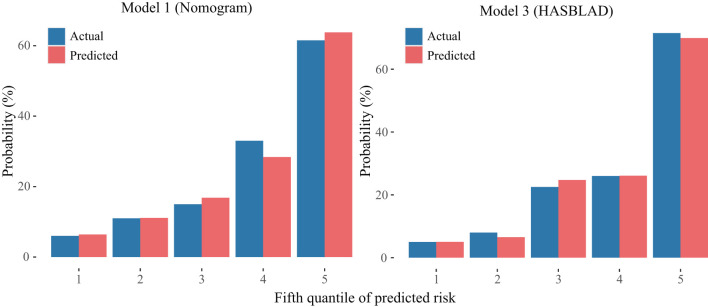
Calibration curves of the Model 1 and the Model 3 in the derivation set. The left plot was the calibration curve of the Model 1 (Nomogram) in the derivation set. The right plot was the calibration curve of the Model 3 (HASBLAD) in the derivation set.

According to the ROC analysis, we draw a preliminary conclusion that both Model 1 (the nomogram; C statistic, 0.781; 95% CI: 0.748-0.813) and Model 3 (HASBLAD score; C statistic, 0.768; 95% CI: 0.735-0.802) performed well in predicting MACEs in the training set. Calibration curves reflected the extent to which a model correctly estimates the absolute risk (whether the values predicted by the model agree with the observed values). The results showed that both Model 1 (the nomogram) and Model 3 (HASBLAD score) predicted the probability of MACEs accurately (Figures 4, 5).

### Baseline Characteristics of the Validation Set

Consistent with training set, cardiac diseases, HT, DM, leukocyte, HGB, AST and general anesthesia still showed a significant difference between MACEs and non-MACEs groups. The age of patients in MACEs group was significantly higher than patients without MACEs (mean age: 67.56 vs. 47.59 years, P < 0.001). Details were showed in Table 6.

**Table 6:** Baseline Characteristics of the validation set.

**Variables**	**Event** **(n = 112)**	**Non-MACEs** **(n = 38,785)**	**P-value**
Age (years)^a^	67.56 ± 14.89	49.59 ± 16.63	<0.001
>70	56 (50%)	4213 (10.9%)	<0.001
Sex (males)	49 (43.8%)	16265 (41.9%)	0.77
General anesthesia	74 (66.1%)	19269 (49.7%)	0.001
Cardiac disease	44 (39.3%)	1112 (2.9%)	<0.001
HT	67 (59.8%)	7965 (20.5%)	<0.001
DM	36 (32.1%)	4090 (10.5%)	<0.001
Leukocyte (×10^9^ l^−1^)^b^	7.91 (5.92, 10.40)	6.73 (5.67, 8.11)	<0.001
HGB (g l^−1^)^b^	119 (102, 133)	138 (128, 152)	<0.001
PLT (×10^9^ l^−1^)^b^	215 (167, 269)	240 (202, 279)	<0.001
ALT (U l^−1^)^b^	19 (11, 27)	20 (15, 26)	0.118
AST > 40 U l^−1^	17 (15.2%)	1282 (3.3%)	<0.001
Fibrinogen (g l^−1^)^a^	3.79 ± 1.25	3.14 ± 0.72	<0.001

a *Mean (SD)*.

b *Median (IOR)*.

*HT, hypertension; DM, diabetes mellitus; HGB, hemoglobin; PLT, platelet; ALT, alanine aminotransferase; AST, aspartate aminotransferase*.

### Internal Validation of the Prediction Model and the Comparison With RCRI

**Figure 6:**
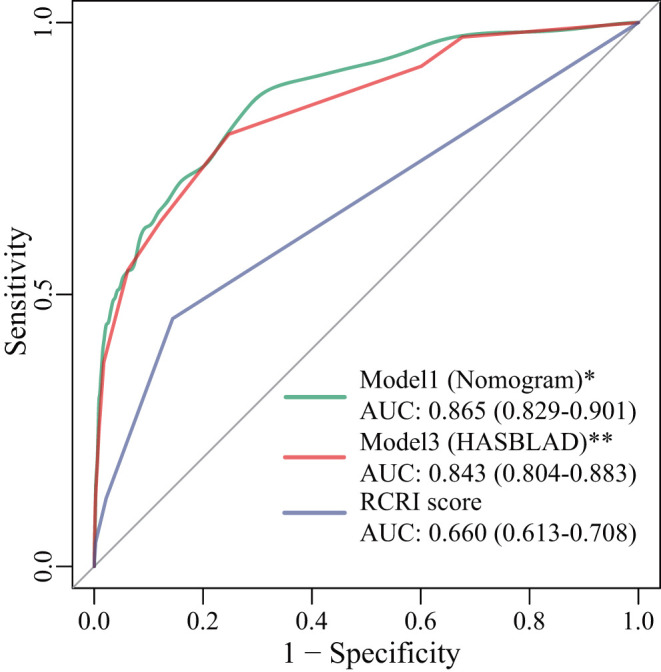
ROC curves for Model 1, Model 3, and RCRI score in the validation set. ^*^: P < 0.001 compared with RCRI score; ^**^: P < 0.001 compared with RCRI score. AUC, area under the curve; RCRI, the revised cardiac risk index; ROC, receiver operating characteristic.

With respect to discrimination in the validation set, Model 1 (the nomogram; C statistic, 0.865; 95% CI: 0.829-0.901) and Model 3 (HASBLAD score; C statistic, 0.843; 95% CI: 0.804-0.883) both performed well in the validation set, while the performance of RCRI (C statistic, 0.660; 95%CI: 0.613-0.708) was relatively poor (Figure 6). The performance of the nomogram and the HASBLAD score was significantly better than RCRI in the evaluation of discrimination (Delong test: P < 0.05). The difference between the nomogram and the HASBLAD score was not statistically significant, suggesting that the predictive performance of the models was comparable (P > 0.05).

**Figure 7:**
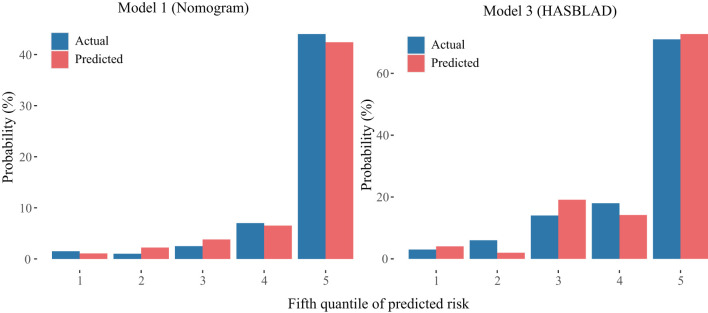
Calibration curves of the Model 1 and the Model 3 in the validation set. The left plot was the calibration curve of the Model 1 (Nomogram) in the validation set. The right plot was the calibration curve of the Model 3 (HASBLAD) in the validation set.

The calibration curve of the nomogram and the HASBLAD score for the probability of MACE both demonstrated good agreement between prediction and observation in the validation set (Figure 7).

### Risk Stratification of Patients in the Validation Set With HASBLAD Score

The incidence of perioperative MACEs was relatively low in our hospital (0.29% in the validation set), and the positive predictive value (PPV) was significantly higher than the observed incidence (Supplementary Tables 3-5). By calculating the prevalence of patients with different scores, we divided patients into low-risk (Points: <4), moderate-risk (Points: 4-7), and high-risk (Points: ≥ 8) groups according to different ranges of HASBLAD score. The risk of MACEs in low-risk, moderate-risk, and high-risk groups were 0.12, 1.23, 14.61%, respectively. Details were showed in Table 7.

**Table 7:** Risk stratification using HASBLAD Score in validation set.

**Points**	**Incidence of MACEs**	**Risk stratification**
<4	0.12%	Low risk
4-7	1.23%	Moderate risk
≥8	14.61%	High risk

*MACEs, major adverse cardiovascular events*.

## Discussion

In recent years, China has witnessed the rapid development of medical technology and reform, with the aging population becoming increasingly prominent. The likelihood of having surgery was quadrupled in the elderly group incorporating cardiovascular diseases, HT, and DM (17). Our research was to target the patients who were likely to suffer perioperative MACEs in all surgical populations. So, the physicians could take measures to reduce perioperative risk promptly for patients in high-risk MACE grading. Risk stratification assists us to be well prepared to respond to emergencies and complications occurred in the perioperative period. On the other hand, timely warning and pre-operative risk information which are included in every pre-operative consultation, will improve the communication consequence between clinicians and patients. It is the main significance of this study. We verified the RCRI score in the validation set, and the result showed its discrimination efficiency was poor (AUC: 0.734; 95%CI: 0.698-0.771), indicating that the differentiation efficiency of the RCRI score may not be superior in Chinese population. Therefore, there is an urgent need for a perioperative cardiac risk model to adapt to the increasing number of patients requiring surgeries in China.

We developed and validated the simple risk assessment tool for the preoperative individualized prediction of MACEs in hospitalized patients who prepared to accept non-cardiac surgery. Our prediction models incorporated seven risk factors, including four clinical factors (general anesthesia, cardiac diseases, DM, and HT), and three laboratory results (HGB, AST, and leukocyte). Current guidelines recommended the use of biomarkers in perioperative evaluation, including N-terminal pro-B type natriuretic peptide (NT-pro BNP), cardiac troponin, and high sensitivity cardiac troponin. However, bringing them into routine screening will cause a serious waste of medical resources. Our model included AST, leukocyte, and HGB which will be screened routinely before operation in China, to evaluate perioperative risk, making full use of medical resources. Many researches have suggested that lower HGB values were associated with myocardial injury or MACEs after non-cardiac surgery (18–20). This was mainly because anemia will damage the ability of the body to compensate for hemodynamic changes, thus organizations were more prone to an imbalance between oxygen supply and demand, leading to an increase in the possibility of type 2 MI (14). Leukocyte played a causal role in the mechanisms of plaque progression, destabilization, erosion or rupture, which will be engendered by acute perioperative stress in major non-cardiac surgery, leading to myocardial injury, MI and stroke (21). Our finding showed that leukocyte was associated with MACEs, improved risk prediction in coronary heart disease patients undergoing non-cardiac surgery, which was in line with previous long-term prospective clinical trials. Both neutrophil to lymphocyte ratio (22) and regulatory T cells (23) were proved to be of additional value for preoperative risk stratification. And the subpopulation of leukocyte could be used as inexpensive and broadly available tools for perioperative risk assessment. Liver was the most important metabolic and detoxification organ in the body. Because the vast majority of operation, included the intermediate-risk and high-risk of MACEs all need the spinal anesthesia or general anesthesia. Most anesthetics are transformed and degraded by the liver. It will decrease liver blood flow of a different degree.

There are sizeable amounts of patients suffered with the clinical condition that asymptomatic hepatic impairment and the hepatitis virus replicates actively, due to high infection rate of Hepatitis B and Hepatitis C viruses in China. Evaluation of pre-operative hepatic function impairment is extremely important, as the extent of perioperative liver injury caused by ischaemia and reperfusion depends primarily on the duration of ischaemia (24) as well as on pre-existing liver diseases. Hepatic ischaemia and subsequent liver dysfunction during perioperative period is associated with a significant deterioration in prognosis (25). Because of the central role of the liver in the metabolic and immunological response to stress, if the physician neglected the impact of liver function on multiple system organs, the consequence will be disastrous. Pre-existing hepatic dysfunction poses a great risk even for non-hepatic surgery, as shown by the higher blood transfusion requirements, longer hospital stays, more complications and increased mortality rate (26).

Elevated AST levels usually indicated liver or myocardium injury. In this study, the result of multivariate regression analysis showed that AST was an independent risk factor for perioperative cardiac events. It meant that liver functional test, especially the indicator of AST had predictive value for perioperative cardiovascular events, which was demonstrated by our study, this is an indicator received less attention in previous studies. If conditions permit, liver function test, especially AST and ALT will be recommended for the vast majority of patients, unless local anesthesia is required and the patient does not have any risk factors in the scoring model.

Both the HASBLAD score and the nomogram performed adequate discrimination in the training set (*C* statistic, 0.781 vs. 0.768) and the validation set (*C* statistic, 0.865 vs. 0.843), which were significantly better than RCRI (*C* statistic, 0.660, *P* < 0.05). More importantly, our clinical models were developed based on patients of Asian origin and the current medical technology in China.

To our knowledge, Takotsubo cardiomyopathy had received insufficient clinical attention and was rarely included in the category of MACEs during the perioperative period. However, according to our preliminary observations and clinical experience, Takotsubo cardiomyopathy also appreciably contributed to the perioperative cardiac events. The clinical manifestations of Takotsubo cardiomyopathy can present as transient regional systolic left ventricular dysfunction (27), decreased ejection fraction, hemodynamic changes, myocardial injury, HF, ventricular arrhythmias, systemic thromboembolism, and cardiogenic arrest (28). The exact pathophysiological mechanisms remained unclear, but many researchers have suggested that the surgery was an important trigger for Takotsubo cardiomyopathy (29). The broader scope of surgical trauma and longer operation time, the more apparent sympathetic activation that patients will encounter, and thereby the incidence of Takotsubo cardiomyopathy will be higher during the perioperative period (30). This cardiovascular complication has received less attention in previous studies, but its incidence was not rare, and the perniciousness could not be ignored in our investigation. Therefore, we included this disease as an outcome event in our study, making the evaluation of perioperative MACEs more comprehensive.

There were some limitations to this study as well. Firstly, it was performed at a single academic medical center and lack of external validation, which may cause deficiencies in representativeness and universality. Secondly, the relatively low incidence of complications might underestimate the actual incidence, especially affected by the lack of routine postoperative surveillance with troponin, NT-pro BNP, and electrocardiography. The biomarker of myocardial injury could assist the physician to recognize the myocardial infarction or HF early before the symptoms flare up. Due to the limitations of medical record-controlled studies, some indicators of great value in the prediction of perioperative cardiovascular events, such as metabolic equivalent, were not recorded routinely in the medical documentation. This reflects the current situation that our physician may have insufficient understanding of the importance of NT-pro BNP, troponin and metabolic equivalent. Considering that MACE has a negative impact on outcome in patients underwent non-cardiac surgery, the awareness of the risk of MACE needs to be strengthened in the management of postoperative patients in Chinese hospitals. Finally, the population number of the validation group was 112 in 2020 and 253 cases of patients from 2010 to 2019, respectively. The relatively fewer quantity of training set was due to the refinement of diagnostic criteria of MACE and standardization of the medical record system. In recent years, the increasing attention to the harmfulness of MACEs in perioperative periods for hospitalized patients also contributes to this phenomenon.

In conclusion, we developed a nomogram and a risk score (HASBLAD) with prediction performance better than RCRI, which could be useful tools at the bedside. The significance of cardiac troponin and NT-pro BNP in perioperative period should be further investigated. At the same time, the focus on Takotsubo cardiomyopathy and the other cardiac complications that caused morbidity and mortality in patients during the perioperative period was necessary and should be further elucidated in future studies.

## Data Availability Statement

The raw data supporting the conclusions of this article will be made available by the authors, without undue reservation.

## Ethics Statement

This study was conducted in accordance with the Declaration of Helsinki and with approval from the Ethics Committee of Peking University Third Hospital (No. M2018258). The patients/participants provided their written informed consent to participate in this study.

## Author Contributions

LZu, WG, and WX put forward the research idea for article. MZ, JC, MX, YX, CW, and HC were responsible for the study implementation and data collection. LZe, YL, and ZS performed the data analysis. MZ and ZS were jointly wrote the article. YF, WX, and LZu critically revised the work. All authors read and approved the final manuscript.

## Funding

This work was supported by the subject research on risk assessment of peri-operative cardiovascular events in artificial joint replacement surgery (grant number BYSY2018014).

## Conflict of Interest

The authors declare that the research was conducted in the absence of any commercial or financial relationships that could be construed as a potential conflict of interest.

## Publisher's Note

All claims expressed in this article are solely those of the authors and do not necessarily represent those of their affiliated organizations, or those of the publisher, the editors and the reviewers. Any product that may be evaluated in this article, or claim that may be made by its manufacturer, is not guaranteed or endorsed by the publisher.
