# Supplementary material for: Development and Validation of Predictive Model—HASBLAD Score—For Major Adverse Cardiovascular Events During Perioperative Period of Non-cardiac Surgery: A Single Center Experience in China
Source: Front Cardiovasc Med. 2022 May 9;9:774191. doi: 10.3389/fcvm.2022.774191 (PMC9124933; doi:10.3389/fcvm.2022.774191)
Supplement: Supplementary file 1 [file Table_1.docx]

Supplementary Appendix

Table S1. Operation type and risk stratification

| Operation type and risk stratification (1) | |
| --- | --- |
| Low risk:  < 1% | Superficial  Breast  Dental  Endocrine: thyroid  Eye  Reconstructive  Carotid asymptomatic (CEA or CAS)  Gynaecology: minor  Orthopaedic: minor (meniscectomy)  Urological: minor (transurethral resection of the prostate) |
| Intermediate risk:  1-5% | Intraperitoneal: splenectomy, hiatal hernia repair, cholecystectomy Carotid symptomatic (CEA or CAS)  Peripheral arterial angioplasty  Endovascular aneurysm repair  Head and neck surgery  Neurological or orthopaedic: major (hip and spine surgery)  Urological or gynaecological: major  Renal transplant  Intra-thoracic: non-major |
| High risk:  > 5% | Aortic and major vascular surgery  Open lower limb revascularization or  amputation or thromboembolectomy  Duodeno-pancreatic surgery  Liver resection, bile duct surgery  Oesophagectomy  Repair of perforated bowel  Adrenal resection  Total cystectomy  Pneumonectomy  Pulmonary or liver transplant |

Abbreviations: CAS: carotid artery stenting; CEA: carotid endarterectomy.

Table S2. The classification of training population according to the type of operation

| Operation type and risk stratification | | MACEs | non-MACEs |
| --- | --- | --- | --- |
| Low risk: | Superficial | 9 | 15137 |
|  | Breast | 0 | 10135 |
|  | Dental | 0 | 252 |
|  | Endocrine: thyroid | 1 | 11750 |
|  | Eye | 1 | 64195 |
|  | Reconstructive | 0 | 4252 |
|  | Carotid asymptomatic (CEA or CAS) | 2 | 663 |
|  | Gynaecology: minor | 1 | 12987 |
|  | Orthopaedic: minor (meniscectomy) | 28 | 53084 |
|  | Urological: minor (transurethral resection of the prostate) | 11 | 11220 |
| total |  | 53 | 183675 |
| Intermediate risk: | Intraperitoneal: splenectomy, hiatal hernia repair, cholecystectomy | 21 | 26270 |
|  | Carotid symptomatic (CEA or CAS) | 10 | 3053 |
|  | Peripheral arterial angioplasty | 17 | 3177 |
|  | Endovascular aneurysm repair | 3 | 24 |
|  | Head and neck surgery | 7 | 11125 |
|  | Neurological | 8 | 7845 |
|  | orthopaedic: major (hip and spine surgery) | 37 | 92494 |
|  | Urological or gynaecological: major | 21 | 52281 |
|  | Renal transplant | 18 | 1150 |
|  | Intra-thoracic: non-major | 3 | 3885 |
| total |  | 145 | 201304 |
| High risk: | Aortic and major vascular surgery | 7 | 568 |
|  | Open lower limb revascularization or amputation or thromboembolectomy | 4 | 7033 |
|  | Duodeno-pancreatic surgery | 6 | 1400 |
|  | Liver resection, bile duct surgery | 3 | 2388 |
|  | Oesophagectomy | 2 | 1454 |
|  | Repair of perforated bowel | 20 | 4654 |
|  | Adrenal resection | 5 | 1532 |
|  | Total cystectomy | 3 | 519 |
|  | Pneumonectomy | 5 | 6830 |
|  | Pulmonary or liver transplant | 0 | 0 |
| total |  | 55 | 26378 |

Abbreviations: CAS: carotid artery stenting; CEA: carotid endarterectomy.

Table S3. Confusion matrix using 4 points (lower value of HASBLAD score) as cut-off value.

|  | Observed | |
| --- | --- | --- |
| Predicted | MACEs | Non-MACEs |
| MACEs | 71 | 4715 |
| Non-MACEs | 41 | 34070 |

Abbreviations: MACEs: major adverse cardiovascular events.

Table S4. Confusion matrix using 8 points (upper value of HASBLAD score) as cut-off value.

|  | Observed | |
| --- | --- | --- |
| Predicted | MACEs | Non-MACEs |
| MACEs | 13 | 76 |
| Non-MACEs | 99 | 38709 |

Abbreviations: MACEs: major adverse cardiovascular events.

Table S5. The analysis of diagnostic efficiencies using different cut-off values of HASBLAD score.

| Cut-off points | Sensitivity | Specificity | PPV | NPV | Prevalence* |
| --- | --- | --- | --- | --- | --- |
| 4 (< 4 points) | 63.40% | 87.84% | 1.48% | 99.88% | 0.29%  0.29% |
| 8 (≥ 8 points) | 11.61% | 99.80% | 14.61% | 99.74% |  |

Abbreviations: PPV: positive predictive value; NPV: negative predictive value; MACEs: major adverse cardiovascular events.

*: The prevalence rate of MACEs in validation set.

Table S6. Baseline characteristics of patients underwent low-risk operation

| Variables | MACEs  (n = 46) | Non-MACEs  (n = 188) | *P*-value |
| --- | --- | --- | --- |
| Age (years) | 71 (58,78) | 70.5 (58,78) | 0.497 |
| Sex (males) | 20 (43.5%) | 80 (42.6%) | 1.000 |
| General anesthesia | 28 (60.9%) | 80 (42.6%) | 0.039 |
| Endoscopic operation | 9 (19.6%) | 32 (17%) | 0.849 |
| Cardiac disease | 13 (28.3%) | 25 (13.3%) | 0.025 |
| HT | 28 (60.9%) | 77 (41%) | 0.023 |
| DM | 11 (23.9%) | 29 (15.4%) | 0.249 |
| CKD | 5 (10.9%) | 3 (1.6%) | 0.008 |
| Length of hospital stay (days) | 14 (9,22) | 9 (5,15) | <0.001 |
| Leukocyte (10^9^/L) | 7.80 (6.23, 10.79) | 6.48 (5.35, 8.10) | <0.001 |
| HGB (g/L) | 120 (102, 136) | 131 (118, 143) | <0.001 |
| PLT (10^9^/L) | 191 (150, 245) | 206 (173 ,255) | 0.001 |
| AST (U/L) | 24 (17, 40) | 21 (17, 25) | <0.001 |
| Scr (umol/L) | 81 (66, 108) | 74 (63, 88) | <0.001 |
| Potassium (mmol/L) | 4.08 (3.80, 4.42) | 4.05 (3.81, 4.30) | 0.258 |
| HASBLAD | 5 (3, 6) | 2 (2, 4) | <0.001 |

Abbreviations HT: hypertension; DM: diabetes mellitus; CKD: chronic kidney disease; HGB: hemoglobin; PLT: platelet; AST: aspartate aminotransferase; Scr: serum creatinine.

**Reference**

1. Kristensen SD, Knuuti J, Saraste A, Anker S, Botker HE, Hert SD, et al. 2014 ESC/ESA Guidelines on non-cardiac surgery: cardiovascular assessment and management: The Joint Task Force on non-cardiac surgery: cardiovascular assessment and management of the European Society of Cardiology (ESC) and the European Society of Anaesthesiology (ESA). *Eur Heart J* (2014) 35(35):2383-431. doi: 10.1093/eurheartj/ehu282.
